# Avoidable emergency department admissions among nursing home residents – insights from a retrospective study

**DOI:** 10.1007/s41999-025-01264-2

**Published:** 2025-07-03

**Authors:** Julie Merche, Henri Thonon, François-Xavier Sibille, Julie Gabriel, Elise Simonin, Didier Schoevaerdts, Thérèse Van Durme, Marie de Saint-Hubert

**Affiliations:** 1 Department of Geriatric Medicine, Centre Hospitalier Universitaire UCL Namur, Namur, Belgium; 2 UCLouvain, Institute of Health and Society, Brussels, Belgium; 3 Emergency Department, Centre Hospitalier Universitaire UCL Namur, Namur, Belgium; 4 UCLouvain, Institute of Experimental and Clinical Research, Brussels, Belgium; 5 Department of Geriatric Medicine, Centre Hospitalier Universitaire Vaudois, Lausanne, Switzerland; 6 UCLouvain, Faculty of Public Health, Brussels, Belgium

**Keywords:** Avoidable emergency admission, Nursing home residents

## Abstract

**Purpose:**

The number of nursing home residents (NHRs) admitted to emergency departments (EDs) is steadily increasing. Some of these admissions have been identified as potentially avoidable. This study aims to quantify avoidable admissions at a Belgian hospital and identify factors associated with these admissions. A retrospective assessment tool is also proposed for the rapid identification of avoidable admissions.

**Methods:**

One-year retrospective descriptive study of NHR admissions to the ED of a Belgian university hospital. The avoidability of each admission was assessed using a retrospective assessment tool and independently assessed by two geriatricians based on clinical judgment. The sensitivity and specificity of the tool were measured against clinical judgment as the reference standard.

**Results:**

A total of 246 NHRs visited the ED, accounting for 327 admissions. Based on clinical judgment, 22.6% of admissions were deemed potentially avoidable. Direct costs were estimated. The retrospective assessment tool identified a rate of 14.1% (specificity: 98.4% and sensitivity: 56.8%). The most common presenting complaints for avoidable admissions were falls (26.1%), minor catheter-related issues (13.5%), and wounds (8.1%). No significant differences were observed in population and NH characteristics between avoidable and non-avoidable admissions.

**Conclusion:**

We identified approximately one in five ED admissions among NHRs as potentially avoidable. This work also presents a specific tool enabling identification and quantification of avoidable admissions. Detecting the most common "avoidable" presenting complaints and diagnoses associated with avoidable admissions presents potential opportunities to explore alternative care solutions within nursing homes.

**Supplementary Information:**

The online version contains supplementary material available at 10.1007/s41999-025-01264-2.

## Key summary points

**Aim:**

To quantify avoidable admissions of nursing home residents in a Belgian hospital, identify associated factors, and propose a retrospective assessment tool.

**Findings:**

Between 14.1% and 22.6% of admissions were deemed potentially avoidable, depending on the assessment method. Falls, catheter issues, and wounds were common causes.

**Message:**

Identifying common avoidable complaints and diagnoses may support the development of alternative care solutions in nursing homes.

**Supplementary Information:**

The online version contains supplementary material available at 10.1007/s41999-025-01264-2.

## Introduction

An increase in emergency department (ED) visits by nursing home residents (NHRs) has been documented in numerous countries [1–5]. For instance, one in four NHRs visits the ED every six months in Canada [6], while an annual rate of 18.9% has been reported in France [7]. These visits are associated with prolonged ED stays, an increased workload for healthcare providers, and rising costs [8]. This trend is multifactorial, driven by the complex healthcare needs of this population [9], care fragmentation, and inadequate primary care support [1, 3]. NHRs frequently present with severe clinical conditions and are at a high risk of hospitalization and mortality [5, 8, 10]. ED visits can pose particular challenges for this demographic due to an unsuitable environment, communication barriers, limited geriatric expertise among emergency healthcare providers, atypical disease presentations, and risks of misdiagnosis, iatrogenesis, health deterioration, and mortality [5, 8–12]. Although most ED admissions for NHRs are deemed necessary, their adverse consequences have prompted efforts to identify potentially avoidable admissions [10]. Reducing avoidable admissions could help mitigate health risks for NHRs and decrease overall healthcare costs [11, 13].

It has been well established that no consensus currently exists regarding the definition of "avoidable" in this context [11, 13]. As such, various terms have been employed, including "avoidable," "preventable," and "inappropriate," often preceded by "potentially," highlighting the complexity of assessment [14]. Reported rates range from 4 to 55%, depending on the context, definition, study design (prospective or retrospective), and measurement tools [2, 11, 15–22]. Additionally, perspectives vary between NHRs, families, caregivers, clinicians, and policymakers [13, 23].

The decision-making process for transferring NHRs to the ED is complex and can lead to admissions being deemed avoidable due to a multitude of factors:

1. Clinical context: The nonspecific nature of health changes in NHRs frequently complicates the presumptive diagnosis [12, 20]. However, certain reasons for transfer may be adequately managed without ED evaluation, either because they are not severe or, as suggested by some authors, because they do not require diagnostic tests [15, 16, 24]. For example, a basic wound requiring only wound care.
2. NHR preferences and advance care planning: Hospital admissions may be avoidable if advance care planning’s have not been established or respected [5, 23, 25], are misaligned with the NHR’s overall condition [16], or cannot be implemented by the nursing home (NH) [26].
3. Family pressures: Family dynamics are particularly relevant in end-of-life situations [23, 26].
4. Nursing home context: Avoidable admissions are associated with factors such as the unavailability of general practitioners (GPs) or specialist consultations [17, 25, 26], staff turnover, staffing levels (which are debated), and staff training [11, 13, 26, 27]. Organizational and communication challenges within NH teams [7, 23, 26, 28] and limitations in diagnostic and treatment resources [13, 14, 16, 26] also contribute to this issue.
5. Healthcare policies: Contributing factors include hospital- and ED-centric care models, a lack of alternative pathways facilitating direct hospital admissions, a safety culture characterized by fear of litigation, economic constraints, and limited geriatric expertise within NHs [3, 13, 20, 23, 26, 29].

To our knowledge, the quantification of avoidable ED admissions for NHRs in Belgium has not been previously documented. Most of studies have been conducted in the USA and Canada, with limited data available from European countries, despite significant differences in healthcare systems [25]. The primary objective of this study is to quantify the annual percentage of avoidable ED admissions for NHRs in a Belgian hospital. Additionally, we evaluate and assess the use of a rapid decision-making tool as a screening method for identifying avoidable admissions and propose actionable recommendations for healthcare providers and policymakers.

## Methodology

### Study setting

The CHU UCL Namur (936 beds, including 300 university care beds and 73,694 annual ED admissions) operates across three hospital sites in the Namur province [30]. This study was conducted at the Godinne site, a rural university hospital. It has been reported in France that rural NHs transfer NHRs to hospitals less frequently than urban NHs [7]. In Belgium, 5% of individuals aged over 65 reside in NHs [29]. The Belgian long-term care system comprises short-stay centres, assisted living residences, residential homes, and nursing homes [31]. In this study, the term *nursing home* is used as a generic designation for all facility types. The level of care covered by public health insurance is determined by care dependency level. Services are delivered by public, non-profit, and for-profit providers, under federal and regional governance [31]. NHRs can visit EDs without prior approval from their GP. They may arrive via personal transportation or private ambulances, both allowing choice of hospital but at the NHR’s expense. Alternatively, public emergency services (via the European emergency number 112) can dispatch an ambulance for a fixed fee, typically transporting the NHR to the nearest hospital. Depending on the situation, a physician-staffed or a nurse -staffed ambulance may also be sent. GPs in Belgium are self-employed and are legally obligated to participate in on-call duties [29].

### Study design and eligibility criteria

Single-centre, descriptive, retrospective study based on data collected from the medical records of all ED admissions involving NHRs aged 70 years or older, between January 1 and December 31, 2022. This study received approval from the hospital's ethics committee in December 2022, with a waiver of informed consent, as data were exclusively obtained from medical records (NUB: B0392025000041).

### Data collection

Two authors (JG and JM) conducted the data collection. ED visits were pseudonymized. Data were manually extracted from ED reports and scanned documents, including letters from GPs or NHs, geriatric letters, hospitalization reports, and records from the belgian health network. Both authors cross-verified the coding and ensured there were no errors. The collected information encompassed sociodemographic data, frailty scores, geriatric scales, comorbidities (including chronic behavioural disorders, classified under “psychogeriatric patient”), and treatments (all medications listed in the Belgian Centre for Pharmacotherapeutic Information compendium, including "pro re nata"). NH characteristics (type, distance from the hospital, dementia units) were also documented. Furthermore, parameters related to ED stays were recorded (timing, mode of transport, decision-maker, length of stay, hospital admission, or discharge home), as well as presenting complaints for admission, in addition to diagnoses, examinations, and administered treatments. A distinction was made between "minor catheter-related issues" (e.g., flushing gastrostomy or cystocath, or urinary catheter placement) and "complex catheter-related issues", which necessitated device replacement by a specialist. Multiple admissions within the year for the same NHR were considered independent events for the assessment of avoidability. Costs were evaluated using the hospital billing service and ambulance cost calculations. Data collection adhered to a written protocol, and any discrepancies were resolved by consensus.

### Outcomes measurement

Defining the avoidability of ED admissions remains a challenge due to the absence of a standardized definition [11]. The systematic review conducted by Renom-Guiteras et al. highlights the heterogeneity of assessment tools and the lack of a comprehensive instrument that incorporates all relevant factors, such as NHR characteristics and the preferences of both NHRs and their families [32]. Similarly, a recent systematic review by Makhmutov et al., which examined assessment tools for avoidable care transitions in older adults, reached a similar conclusion: existing instruments lack comprehensiveness, as they address only a limited number of perspectives [33]. Two primary approaches are commonly utilized:

1. The first approach relies on the concept of "ambulatory care sensitive conditions" (ACSCs) and defines avoidable admissions as those resulting from a failure of preventive care [2, 6, 18, 21, 34, 35]. This list of conditions (e.g., heart failure, chronic obstructive pulmonary disease), has been established, and these diagnoses can be easily identified using International Classification of Diseases and Related Health Problems (ICD-10) codes, which can be automatically extracted from databases retrospectively [2, 34].
2. The second approach focuses on admissions involving “low-acuity” visits that do not necessitate ED management and could instead be safely handled within the NH [6, 16, 17, 19, 36]. These visits typically encompass low-severity diagnoses (e.g., minor wounds, uncomplicated urinary tract infections, and mild dehydration) which exhibit low triage urgency and result in frequent discharges back to the NH [6, 9, 35]. Such admissions often reflect deficiencies in resources, medical expertise, or diagnostic capabilities within NHs. Their assessment is based on various methods, including chart reviews guided by explicit criteria, expert judgment regarding the necessary level of care, and/or structured tools such as numerical appropriateness scales, structured implicit review forms, or the Appropriateness Evaluation Protocol [2, 15–18, 20, 22, 37, 38].

We opted for the term "avoidable" rather than "inappropriate". Working retrospectively, not all perspectives or contextual factors could be collected. Indeed, it was thus less feasible to state about «failure of preventive care», which limits the appropriateness of using the term "inappropriate." [15]. We therefore focused on low-acuity visit more than on ACSCs.

**Fig. 1:**
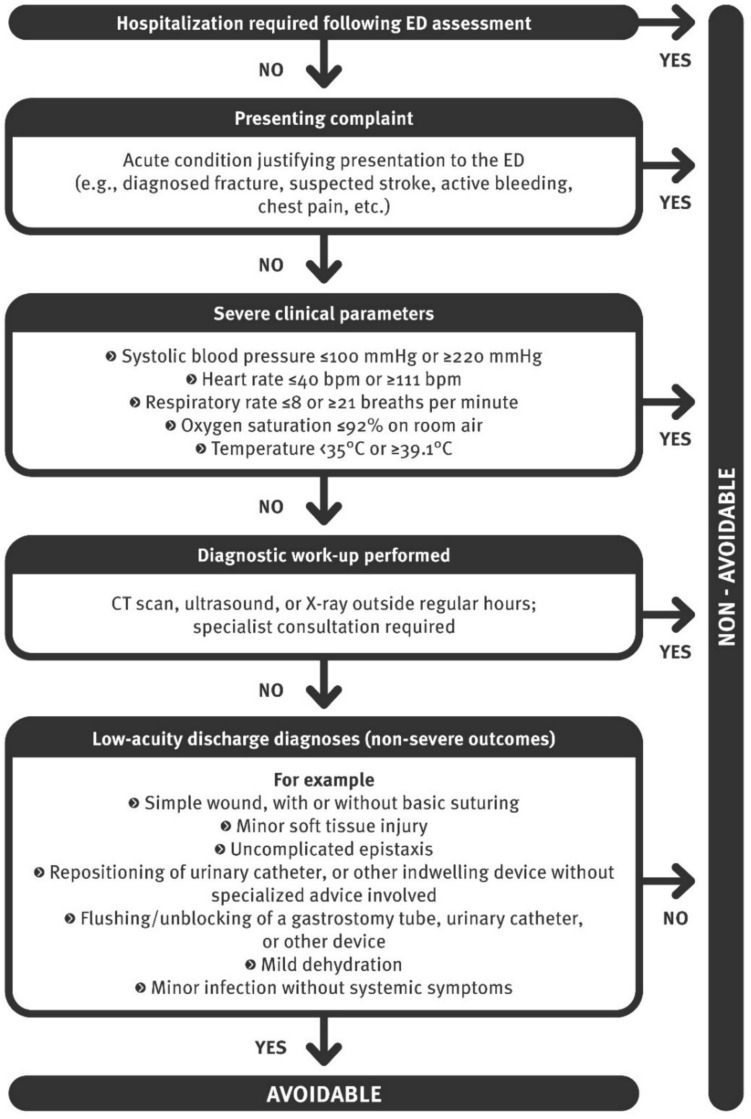
Retrospective assessment tool. ED, emergency department; CT, Computed tomography

Two approaches were employed in our analysis: (1) a retrospective assessment tool (Fig. 1) designed to determine the avoidability of ED admissions among NHRs, based on criteria derived from the literature and retrospectively available in medical records, developed by the authors. Additional file 1 presents these criteria. (2) an independent clinical judgment by two geriatricians with heterogeneous backgrounds: one senior physician who had led the literature review on the concept of avoidable hospital admissions, and one junior physician in her final year of geriatric training, conducting a master's thesis on the topic. They independently answered the question, “Was this admission avoidable?” without being influenced by the NHR 's outcome.

The evaluations were conducted independently of the retrospective assessment tool and of each other. Disagreements were resolved through consensus, and the reproducibility of the judgments was analysed. This dual approach, combining an explicit tool with subjective clinical judgment, takes into account implicit and contextual factors [16, 32, 37].

Lastly, we calculated the costs based on the hospital billing records for each visit considered avoidable. The calculation of ambulance costs also took into account the distance travelled, and the pricing depending on whether the intervention involved the 112-emergency service or not.

### Statistical analysis

Quantitative variables were presented as mean (SD – standard deviation) for normal distributions or as median (first and third quartile [Q1–Q3]) for non-normal distributions, as determined by the Shapiro–Wilk test. Categorical variables were analysed using the Chi-squared test of independence or Fisher's exact test. Quantitative variables were compared utilizing Student’s t-test or the Mann–Whitney U test, depending on the data distribution. The threshold for statistical significance was established at 5%. Inter-rater agreement was assessed using Cohen’s kappa coefficient. No records were excluded, even in the presence of missing data. All analyses were conducted using R software, version 4.3.1 (2023–06-16). Variables unrelated to the ED were analyzed using univariate binomial logistic regression to evaluate their potential in predicting the risk of avoidable admission. Variables demonstrating a significant association (*p* < 0.10) were subsequently included in the multivariate regression. A stepwise regression approach, incorporating both forward and backward selection, was employed to optimize the model’s Akaike Information Criterion and identify independent predictors of avoidable admission.

## Results

### Population and NHs description

In 2022, a total of 16,476 visits to the ED were recorded, of which 4,057 involved patients aged over 70 years (24.6%). Among these, 327 visits were attributed to NHRs, accounting for 8.1% of ED visits in the > 70 age group. The 327 admissions involved 246 NHRs, with 20.7% presenting multiple times throughout the year (See Additional file 2). The characteristics of the study population are summarized in Table 1. The median age was 86 years [81–90], and 67.9% of the population were women. The NHRs exhibited a high level of frailty: a median Clinical Frailty Scale score of 7 [6, 7] and a median Katz index of 15 [11–20]. Additionally, 57.3% required assistive devices for mobility, 19.0% were bedridden, and the median Charlson Comorbidity Index was 7 [6–8]. Furthermore, 55.7% of the NHRs had moderate to severe major neurocognitive disorders, 25.7% presented with behavioural disorders. Polypharmacy was prevalent, with a median of 11 medications [8–14]. NHRs were admitted from 51 NHs, of which 11 (21.6%) were public, 19 (37.3%) were private for-profit facilities, and 21 (41.2%) were non-profit associations. Among these facilities, 82.3% offered multiple services (assisted living residences, residential homes, and nursing homes), while 11.8% provided only beds under the status of residential homes, and 5.9% were classified solely as assisted living residences. Eleven NHs (21.6%) had a dedicated protected unit for NHRs with cognitive impairment. The median distance between the NHs and the hospital was 28 km [17.1–41.9], which corresponded to a median travel time of 32 min [20.5–40.5].

**Table 1:** population and nursing homes characteristics

	Non-avoidable^a^ (*N* = 253)	Avoidable^a^ (*N* = 74)	All *N* = 327	*p*-value
**Age**, y	86 [81–91]	87 [81–89]	86 [81–90]	0.987
**Women**	178 (70.4%)	44 (59.5%)	222 (67.9%)	0.104
**ISAR**	4 [3, 4]	4 [3, 4]	4 [3, 4]	0.904
*Missing*	37 (14.3%)	20 (27.0%)	57 (17.4%)	
ISAR at 0 or 1: low risk	0 (0.0%)	1 (1.9%)	1 (0.4%)	0.200
ISAR greater than or equal to 2: at risk	216 (100.0%)	53 (98.2%)	269 (99.6%)	
**Clinical Frailty Score**	7 [6, 7]	7 [6, 7]	7 [6, 7]	0.941
*Missing*	10	3	13	
1 to 4: Very Fit to vulnerable	0 (0%)	0 (0%)	0 (0%)	0.678
5: Mildly Frail	9 (3.7%)	1 (1.4%)	10 (3.2%)	
6: Moderately Frail	71 (29.2%)	24 (33.8%)	95 (30.3%)	
7: Severely frail	156 (64.2%)	43 (60.6%)	199 (63.4%)	
8: Very severely Frail	6 (2.5%)	3 (4.2%)	9 (2.9%)	
9: Terminally	1 (0.4%)	0 (0.0%)	1 (0.3%)	
**Katz Score** (/24)	15 [11–20]	15 [12.2–20]	15 [11–20]	0.459
*Missing*	60 (23.7%)	20 (27.0%)	80 (24.5%)	
**Mobility**				0.766
Bedridden	49 (19.8%)	11 (15.9%)	60 (19.0%)	
Dependant on assistive device	140 (56.7%)	41 (59.4%)	181 (57.3%)	
Independent	58 (23.5%)	17 (24.7%)	75 (23.7%)	
*Missing*	6	5	11	
**Number of medications on the treatment list**	11 [8–14]	11 [7–13]	11 [8–14]	0.340
**Central nervous system acting drugs** (number)	2 [1–3]	2 [1, 2]	2 [1–3]	0.075
**Charlson** (/37)	7 [6–8]	7 [6–9]	7 [6–8]	0.619
** Age**				0.963
70–79 years	61 (24.1%)	17 (23.0%)	78 (23.9%)	
≥ 80 years	192 (75.9%)	57 (77.0%)	249 (76.2%)	
** Myocardial infarction**				> 0.999
Yes	68 (26.9%)	20 (27.0%)	88 (26.9%)	
** Congestive Heart Failure**				0.651
Yes	88 (34.8%)	23 (31.1%)	111 (33.9%)	
** Peripheral vascular disease**				0.241
Yes	47 (18.6%)	19 (25.7%)	66 (20.2%)	
** Cerebrovascular Accident or Transient Ischemic Attack**				0.703
Yes	74 (29.3%)	24 (32.4%)	98 (30.0%)	
** Dementia**				0.855
Yes	142 (56.1%)	40 (54.1%)	182 (55.7%)	
** Chronic Obstructive Pulmonary Disease**				0.410
Yes	69 (27.3%)	16 (21.6%)	85 (26.0%)	
** Connective tissue disease**				> 0.999
Yes	10 (3.9%)	3 (4.1%)	13 (4.0%)	
** Peptic ulcer disease**				0.895
Yes	88 (34.8%)	27 (36.5%)	115 (35.2%)	
** Liver disease**				0.636
Mild	7 (2.8%)	3 (4.1%)	10 (3.1%)	
Moderate to severe	7 (2.8%)	3 (4.1%)	10 (3.1%)	
** Diabetes mellitus**				0.176
Uncomplicated	22 (8.7%)	8 (10.8%)	30 (9.2%)	
End-organ damage	26 (10.3%)	13 (17.6%)	39 (11.9%)	
** Hemiplegia**				0.693
Yes	23 (9.1%)	5 (6.8%)	28 (8.6%)	
** Moderate to severe chronic kidney disease**				0.357
Yes	7 (2.8%)	0 (0.0%)	7 (2.1%)	
** Solid tumor**				0.299
Localized	19 (7.5%)	10 (13.5%)	29 (8.9%)	
Metastatic	14 (5.5%)	4 (5.4%)	18 (5.6%)	
** Leukaemia**				> 0.999
Yes	5 (1.9%)	1 (1.4%)	6 (1.8%)	
** Lymphoma**				0.717
Yes	8 (3.2%)	3 (4.1%)	11 (3.4%)	
** AIDS**				
Yes	0	0	0	
**Psychogeriatric patient**	65 (25.7%)	19 (25.7%)	84 (25.7%)	> 0.999
**Nursing homes characteristics**				
Private for-profit facilities	98 (38.7%)	36 (48.7%)	134 (41.0%)	0.164
Non-profit associations	120 (47.4%)	30 (40.5%)	150 (45.9%)	0.361
Public	39 (15.4%)	10 (13.5%)	49 (15.0%)	0.827
Total number of beds	144 [124–170]	150 [92.5–182]	144 [124–170]	0.852
Alzheimer specific unit	91 (36.0%)	30 (40.5%)	121 (37.0%)	0.562
Distance from ED, km	11.5 [3–22.3]	11.1 [3.0–22.3]	11.5 [3.0–22.3]	0.626
Distance from ED, min	15 [6–25]	14 [6–25]	15 [6–25]	0.638

ISAR, Identification of Seniors At Risk; AIDS, Acquired immunodeficiency syndrome; NH, nursing home; ED, emergency department

Value are mean ± SD, n (%) or median [p25-p75]

^a^Avoidable or not based on the assessment of two geriatricians. The analyses were conducted both by considering uncertain cases as non-avoidable and by treating them as a separate group. The observed differences remained statistically significant when categorizing admissions as avoidable or not based on the retrospective assessment tool

### ED admission description

The characteristics of ED visits are outlined in Table 2. Regarding transport, 88.6% arrived by ambulance, comprising 49.5% using emergency medical services, 21.7% arriving by private ambulance, and 17.4% through physician-staffed ambulance. In the cohort, 52.9% were referred by their GP (either via telephone or following a consultation). The primary presenting complaints for admission included falls and suspected fractures (21.0%), respiratory symptoms (9.3%), and confusion (8.1%). The most common diagnoses were fractures or functional decline (15.1%), delirium (7.2%), and pneumonia (6.2%). Additional diagnostic tests were performed in 92.4% of cases. Among the NHRs, 23 (7.0%) had a care plan classified as palliative, of whom 21 had underwent at least one diagnostic test. The median duration of stay in the ED was 304 min [233–411], and 43.1% of NHRs returned to their NH following the ED visit.

**Table 2:** Characteristics of ED visits

	Non-avoidable^a^ (*N* = 253)	Avoidable^a^ (*N* = 74)	All *N* = 327	*p*-value
**Day of admission**				0.08
Weekend, public holiday or overnight	89 (35.2%)	18 (24.3%)	107 (32.7%)	
Business day	164 (64.8%)	56 (75.7%)	220 (67.3%)	
Monday	32 (12.7%)	16 (21.6%)	48 (14.7%)	0.083
Tuesday	30 (11.9%)	8 (10.8%)	38 (11.6%)	0.967
Wednesday	21 (8.3%)	7 (9.5%)	28 (8.6%)	0.938
Thursday	31 (12.3%)	15 (20.3%)	46 (14.1%)	0.120
Friday	49 (19.4%)	10 (13.5%)	59 (18.0%)	0.327
**Length of stay in the ED**, min	324 [249–424]	245 [146–302]	304 [233–411]	** < 0.001**
**Mode of transportation**				
Private ambulance	49 (19.4%)	22 (29.7%)	71 (21.7%)	**0.011**
Emergency medical service (112)	127 (50.2%)	35 (47.3%)	162 (49.5%)	
Physician-staffed ambulance (112)	52 (20.6%)	5 (6.8%)	57 (17.4%)	
Personal transportation	25 (9.9%)	12 (16.2%)	37 (11.3%)	
**Who refers the patient to the ED?**				0.709
Patient or relatives	4 (1.6%)	2 (2.7%)	6 (1.8%)	
NH	113 (44.7%)	35 (47.3%)	148 (45.3%)	
GP or on-call GP	136 (53.8%)	37 (50,0%)	173 (52.9%)	
**Transition of care letter from the NH**	203 (80.2%)	48 (64.9%)	251 (76.8%)	**0.009**
**Referral letter from the GP**	106 (41.9%)	30 (40.5%)	136 (41.6%)	0.941
**Pre-existing and established palliative care plan before admission**	17 (6.7%)	6 (8.1%)	23 (7.0%)	0.879
**Assessment conducted in the emergency department**	248 (98.0%)	54 (73.0%)	302 (92.4%)	** < 0.001**
**Blood test**	233 (92.1%)	44 (59.5%)	277 (84.7%)	** < 0.001**
**Arterial blood gas analysis**	90 (35.6%)	2 (2.7%)	92 (28.1%)	** < 0.001**
**Blood cultures**	75 (29.6%)	4 (5.4%)	79 (24.2%)	** < 0.001**
**Urine culture sediment**	117 (46.3%)	15 (20.3%)	132 (40.4%)	** < 0.001**
**COVID-19 swab**	82 (32.4%)	6 (8.1%)	88 (26.9%)	** < 0.001**
**X-ray**	172 (68.0%)	30 (40.5%)	202 (61.8%)	** < 0.001**
**CT scan, ultrasound, MRI**	134 (53.0%)	20 (27.0%)	154 (47.1%)	** < 0.001**
**Electrocardiogram**	184 (72.7%)	22 (29.7%)	206 (63.0%)	** < 0.001**
**Specialist consultation required**	223 (88.1%)	23 (31.1%)	246 (75.2%)	** < 0.001**
**Treatment administered in the ED**				
Oral	65 (25.7%)	20 (27.0%)	85 (26.0%)	0.936
Intravenous	174 (68.8%)	13 (17.6%)	187 (57.2%)	** < 0.001**
Aerosols	24 (9.5%)	1 (1.4%)	25 (7.7%)	**0.039**
Oxygen therapy	57 (22.5%)	2 (2.7%)	59 (18.0%)	** < 0.001**
**Wound suturing**	5 (2.0%)	5 (6.8%)	10 (3.1%)	0.051
**Patient spent the night in the ED**	28 (11.1%)	6 (8.1%)	34 (10.4%)	0.605
**Patient died during the ED stay**	2 (0.8%)	0 (0.0%)	2 (0.6%)	> 0.999
**Discharge destination from the ED**				** < 0.001**
Hospitalization	177 (70.0%)	7 (9.5%)	184 (56.3%)	
Nursing home	74 (29.3%)	67 (90.5%)	141 (43.1%)	
Death	2 (0.8%)	0 (0.0%)	2 (0.6%)	
**Patient refuses hospitalization**	4 (1.6%)	4 (5.4%)	8 (2.5%)	0.081
**Hospitalization within 10 days prior**	16 (6.32%)	11 (14.86%)	27 (8.26%)	**0.035**

ED, emergency departement; NH, nursing home; GP, general practionner; COVID-19, coronavirus disease; CT-scan, Computed tomography; MRI, Magnetic resonance imaging

Value are mean ± SD, n (%) or median [p25-p75]

Values in bold indicate statistically significant *p*-values (*p* < 0.05)

^a^Avoidable or not based on the assessment of two geriatricians. The analyses were conducted both by considering uncertain cases as non-avoidable and by treating them as a separate group. The observed differences remained statistically significant when categorizing admissions as avoidable or not based on the retrospective assessment tool

### Avoidable admissions

**Fig. 2:**
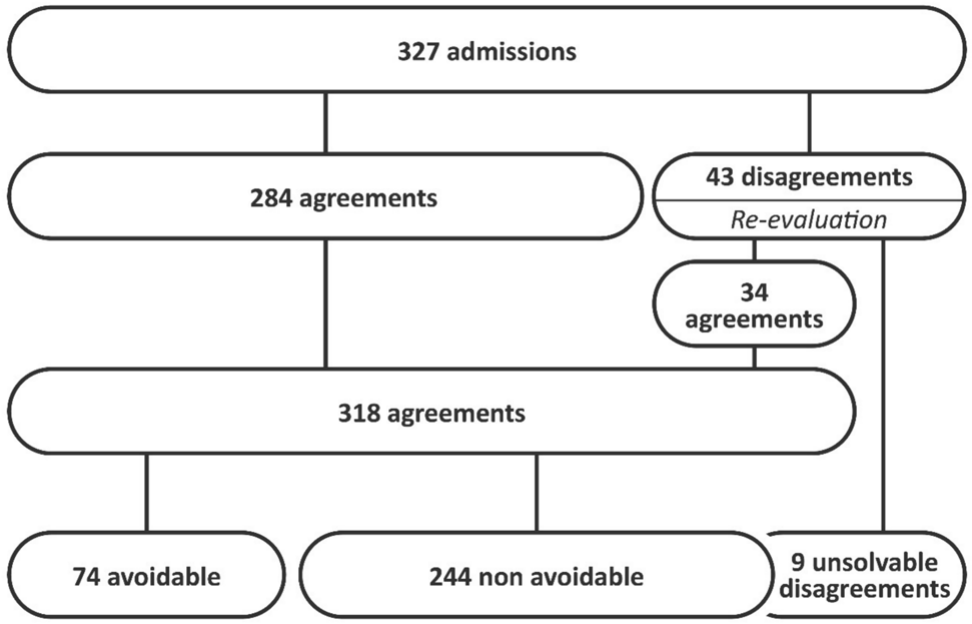
Agreement – disagreement: flow chart. At first review, the two geriatricians agreed on 284 cases and disagreed on 43. After re-evaluation, 9 disagreements remained. These 9 cases were considered non-avoidable for the purpose of analysis

According to the clinical judgment of two geriatricians, 22.6% of admissions were deemed avoidable, with an inter-rater agreement of 86.9% (substantial agreement, Cohen’s kappa: 0.66). Disagreements in clinical judgment were noted for 43 cases (13.2%), of which consensus was reached for 34 cases following review (79.1%), while 9 cases remained unresolved due to insufficient detail/information (Fig. 2). These unresolved cases were subsequently classified as non-avoidable. The retrospective assessment tool yielded a more conservative avoidability rate of 14.1%. This tool analysis demonstrated a specificity of 98.4% and a sensitivity of 56.8%, with an Area Under the Curve (AUC) of 0.776 (95% CI: 0.71–0.89). High positive and negative predictive values were obtained at 91.3% and 88.6% respectively.

No significant differences were observed in population characteristics based on whether the admissions were deemed avoidable or not. We were unable to demonstrate any association between NH characteristics and the avoidability of admissions (Table 1).

**Fig. 3:**
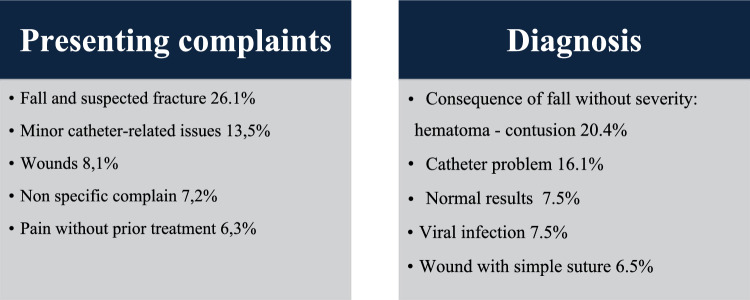
Presenting complaints and Diagnoses in Avoidable Cases

Avoidable admissions were associated with shorter ED stays, the absence of diagnostic tests, and a higher frequency of discharges back to the NH. No significant differences were observed regarding the timing of admission (weekdays vs. nights/holidays) or the referring individual. However, a large part of avoidable admissions were transported by ambulance (83.8%), with 64.5% of them arriving under the regulation of the 112 european emergency number. Avoidable admissions were less likely to include a transfer letter from the NH. The most frequent presenting complaints and diagnoses for avoidable admissions are summarized in Fig. 3. Prominent presenting complaints include falls, minor catheter-related issues, and wounds.

### Costs

The total cost of the 74 avoidable admissions, calculated based on hospital billing (including examinations and monitoring fees), amounted to €14,624.81. The median cost per avoidable admission was €136.94 [€96.59–€209.04], with a range from €45.26 to €888.63. The costs related to ambulance transportation amounted to €8,615.77. This amounts to a total of €23,240.58, of which nearly 40% corresponds to transportation costs, borne by the patient.

### Predictors of avoidable admission

The variables significantly associated with avoidable ED admissions according to clinical judgment, independent of emergency department-related factors, are summarized in Table 3. In the univariate analysis, the timing of admission, mode of transport, and presence of an explanatory letter from NH influenced the likelihood of avoidable admission. Additionally, recent hospitalization within the previous 10 days, refusal of hospitalization, and an increased number of central nervous system-acting drugs were also linked to avoidability. Among the most common presenting complaints for transferring NHRs, falls with suspected fractures, catheter-related issues, wounds, and pain without prior treatment attempts were all associated with avoidable ED admissions. In the multivariate analysis, five variables emerged as independent predictors of avoidable admissions: hospitalization within 10 days prior, pain without truing a treatment, falls with suspected fractures, admission on Mondays, and ambulance transport. The area under the operating curve ROC (receiver operating characteristic) of the multivariate model is 0.794 [95% CI: 0.697–0.892].

**Table 3:** Univariate and multivariate analysis of avoidable admission

	Univariate regression			Multivariate regression		
	OR	95% CI	*P* value	OR	95% CI	*P* value
Minor catheter-related issues	9.948	3.929–25.189	< 0.001			
Wound	4.705	1.806–12.257	0.002			
Transition of care letter from the NH	0.455	0.257–0.803	0.007			
Physician-staffed ambulance	0.28	0.108–0.73	0.009			
Number of Central nervous system acting drugs, by 1	0.816	0.663–1.004	0.054			
Admission on weekend, public holiday or overnight	0.582	0.323–1.05	0.072			
Patient refuses hospitalization	3.557	0.868–14.585	0.078			
Hospitalization within 10 days prior	2.586	1.143–5.851	0.023	6.113	1.125–33.216	0.036
Pain without prior treatment	6.065	1.99–18.484	0.002	4.825	0.85–27.398	0.076
Fall and suspected fracture	3.133	1.561–6.288	0.001	3.512	1.149–10.732	0.027
Admission on Mondays	1.905	0.979–3.709	0.058	3.183	0.963–10.524	0.058
Private ambulance transport	1.761	0.978–3.171	0.059	2.992	0.999–8.962	0.05

NH, nursing home

## Discussion

This study presents the first quantitative evaluation of potentially avoidable ED admissions among NHRs in Belgium. Using a dual-assessment approach–combining structured clinical judgment and a retrospective assessment tool–we found that approximately 22.6% of ED visits could be considered avoidable, with a more conservative estimate of 14.1% derived from the retrospective assessment tool (consistent with its stringent criteria). These results fall within the range reported in international literature (4% to 55%) and underscore the influence of methodological variability, inconsistent definitions, healthcare system structures, and differing perspectives [2, 11, 13, 15–23]. More specifically, our rate of avoidable admissions aligns within the range reported in other studies utilizing the definition of low-acuity visits, i.e., transfers that could have been managed within the NH [6, 9, 15, 17, 22, 38].

Our choice of the term "avoidable" rather than "inappropriate" reflects the retrospective nature of our analysis and acknowledges the multifactorial, context-dependent decision-making process surrounding ED transfers. This aligns with existing literature that highlights the complexity and subjectivity of avoidability assessments, especially when multiple stakeholders are involved [13, 32, 33].

Avoidable admissions in our cohort were characterized by less severe clinical presentations, shorter ED stays, fewer diagnostic procedures, and a higher likelihood of discharge back to the NH. The most frequent presenting complaints included falls and suspected fractures, minor catheter-related issues, wounds, non specific complain and pain without prior treatment – conditions that could likely have been managed on-site given adequate resources and clinical support. These findings reinforce concerns raised in earlier studies regarding the limitations in diagnostic capacity, staff expertise, and clinical decision-making confidence within NHs [6, 9, 36].

Multivariate analysis identified five independent predictors of avoidable admissions: hospitalization within 10 days prior, pain without truing a treatment, falls with suspected fractures, admission on Mondays, and ambulance transport. These variables reflect both systemic and organizational weaknesses. For example, Monday admissions may stem from delayed decisions over the weekend, while recent hospitalizations suggest possible premature discharges or inadequate post-discharge planning. The frequent use of ambulance transport for low-acuity conditions also highlights the need to revisit triage protocols and develop alternative care pathways.

Interestingly, structural features of NHs–such as facility type, profit status, specialized Alzheimer’s units, and distance from the hospital–were not associated with avoidable admissions. Prior literature has noted that Alzheimer’s units and public facility status can influence transfer rates [17, 18]. The distance from the hospital also showed no clear relationship with avoidability, consistent with mixed findings in existing studies [18]. Our results align with past studies suggesting that such transfers are more influenced by internal practices, care culture, and staff capacity than by institutional characteristics [11, 13, 23, 26].

From an economic standpoint, the avoidable admissions in this study resulted in direct hospital costs and transportation cost exceeding €23,000 annually–excluding indirect costs. Cost evaluation remains challenging, as not all procedures performed during avoidable admissions are unnecessary. Some NHRs may still require diagnostic evaluations outside the ED, and ambulance use may be unavoidable in certain clinical contexts. While modest on a per-facility basis, this figure underestimates the broader societal impact when scaled to the national level. These findings highlight the potential for significant cost savings and improved care outcomes through better management of NHRs prior to ED admission [8, 11, 13].

The study population displayed high levels of frailty, multimorbidity, cognitive impairment, and polypharmacy, consistent with previous literature [9, 18, 39]. However, no specific NHR -level characteristics were independently predictive of avoidability. While factors like age, dementia, and indwelling devices use have been associated with ED transfers, they are insufficient for identifying avoidable admissions [4, 34, 40]. Our findings instead point to situational and organizational factors as key drivers.

Most ED visits, including avoidable ones, occurred during working hours, with a peak on Fridays, as observed by others [3, 10, 16, 18, 36, 40]. This contradicts the assumption that inappropriate transfers mainly occur during weekends or nights due to reduced staffing [29] and suggests that system-level improvements–such as enhanced access to diagnostics and treatment during working hours–could reduce ED admission [3, 8, 36]. NHRs are mainly transported by ambulance, and medically-assisted transport is less frequently avoidable–a finding also reported by other authors [2, 3, 8, 10, 18, 40].

Notably, half of the transfers occurred without prior GP consultation, often based on NH staff decisions. Although clinical assessments by GPs are known to reduce avoidable transfers [19, 41], such assessments were not consistently documented in our cohort. This has been reported reflecting either the need for a prompt decision [18] or challenges in reaching the GP [40]. We did not assess the involvement of NHRs or families in the decision-making process, though literature suggests that such involvement is often minimal [42]. Finally, consistent with the findings from other studies, we did not observe a significant difference in the appropriateness of admissions based on the decision-maker [18].

Among the 327 visits, 43.1% of NHRs were discharged back to their NHs, and nearly half of these visits were considered avoidable. Nevertheless, an ED avoidable admission may still result in hospitalization, especially when clinical uncertainty, fear of under-treatment, or medicolegal concerns drive decisions [11, 16, 29].

In 92.4% of cases, diagnostic tests were performed–even in 73.0% of avoidable cases. However, the performance of an additional examination does not necessarily justify an ED admission [36], particularly in cases involving routine blood tests that could be conducted during office hours by the GP. In Belgium, patients can access basic X-rays and blood tests without going through the ED. Therefore, we concluded that the need for a simple examination available during working hours does not, by itself, warrant ED admission. Advanced imaging (CT-scans (Computed tomography) and ultrasounds), can also be accessed rapidly through an emergency referral by the GP; however, this process entails taking time to initiate the request and may involve a delay in availability. Our findings also underscore the challenges related to accompanying NHRs to medical examinations. In many cases, ED admissions could have been managed on an outpatient basis with the presence of someone to accompany the NHR. The use of the ED sometimes reflects a lack of logistical support for scheduled diagnostic procedures, leading to hospital admission for practical rather than clinical reasons. Nonetheless, some examinations performed during avoidable visits reflect concerns regarding litigation and an overly stringent application of the duty of care [3]. For instance, 27.0% of avoidable admissions involved advanced imaging. These findings are consistent with previous studies, particularly concerning cranial CT-scans performed on NHRs who were subsequently discharged [24].

Falls were the most frequent presenting complaint for admission–including among avoidable visits–highlighting their clinical and organizational complexity in NHs. Although most falls are minor [24, 36], some result in serious injuries requiring emergency care (e.g., fractures, cranial trauma) [43]. It is well known that the absence of clear history, heteroanamnesis, or NHR communication can complicate triage decisions [43, 44]. Within our cohort, falls classified as avoidable admissions were often associated with basic assessments (such as X-rays and blood tests), prompting questions regarding the necessity of ED admission. Effective fall management requires a combination of preventive strategies (e.g. medication reviews, environmental adaptations), decision-making algorithms to support NH caregivers, access to radiology services, and enhanced monitoring capabilities within NHs [16, 24, 36]. In Norway, for instance, mobile imaging services prevented up to 70% of transfers [45], although logistical barriers such as staffing and prescribing authority remain concerning [46].

Catheter-related issues were the second leading cause of avoidable transfers–a known risk factor for unnecessary ED use [4, 34, 35, 47]. Many of these issues involved minor procedures (e.g., flushing or replacement) that could be performed in NHs at the condition of better staff training and material provision [40].

Advance care planning was rarely documented in our cohort, despite its known role in reducing inappropriate care transitions [11, 19, 34]. Additionally, we observed a non-significant trend toward a higher frequency of hospitalization refusals in the avoidable admissions group. In Belgium, advance care planning is often incomplete or misaligned with NHR wishes [29], contributing to transfers that may not reflect care preferences. While palliative care plans were present in some cases, they do not necessarily include "do not hospitalize" directives and therefore did not always lead to classification as avoidable. Indeed, hospital admission is not always preferred in cases of imminent death, but nevertheless is not prohibited when symptoms are difficult to manage [16]

A major strength of our study lies in its methodology: data were rigorously collected and cross-validated by two authors, with high inter-rater agreement. The novel retrospective assessment tool, designed for simplicity and reproducibility, offers a practical tool for identifying avoidable admissions in real-time. We did not utilize the Structured Implicit Review tool, which requires unavailable retrospective data [16], nor the Appropriateness Evaluation Protocol (AEP), which tends to overestimate avoidable admissions [18]. Our goal is to provide a conservative, user-friendly tool that informs policymakers on strategies to reduce avoidable admissions, thereby minimizing the risk of misclassifying an admission as avoidable, which could restrict ED access for NHRs or compromise care [5, 14]. We did not investigate ambulatory care-sensitive conditions, which are often linked to severe outcomes, high hospitalization rates, and increased mortality [6]. Reducing avoidability to a single diagnosis oversimplifies transfer decisions [14, 23], which must consider severity, NHR needs, and NH capabilities [13, 14, 39]. The retrospective assessment tool is designed to quickly identify avoidable admissions without claiming to be exhaustive. Significant variability in avoidability assessments has been reported when different caregiver profiles are involved [20, 22, 48]. For instance, NH staff often estimate a lower number of transfers as avoidable [23]. Finally, the study provides the first quantification of this issue in Belgium.

However, several limitations must be acknowledged. The main limitation lies in the retrospective assessment of avoidability, combined with the single-center design and a limited patient sample. Indeed, the retrospective design limits our ability to capture contextual or psychosocial elements of the decision-making process. Our study included only NHRs who ultimately reached the ED, excluding potential under-referrals. Additionally, the lack of NH-level data prevented deeper insight into organizational practices and staff decision-making frameworks–an issue common to many clinician-led studies [14, 40]. Finally, while clinical judgment was used to enrich our analysis, it remains subjective and may vary across professional profiles [20, 22, 48].

## Perspective

The findings of this study underscore several actionable strategies that may help reduce avoidable ED transfers among NHRs.

Firstly, improving access to outpatient diagnostic services, such as radiological imaging and routine blood testing, could enable more timely on-site management of common conditions–particularly those related to falls–thus reducing the need for ED attendance [20, 48].

Secondly, enhanced training for NH staff in procedures such as intra-veinous therapy, catheter management and basic wound care may empower them to manage low-acuity conditions without requiring hospital transfer [11, 20, 36, 48]. Some studies have explored hospital-at-home models in NHs. Kunkel et al. found that timely intravenous therapy reduced ED admissions [50]. In De Stampa et al., NH residence was associated with a lower 30-day readmission risk after hospital-at-home care involving notably intravenous therapy [51]. Expanding staff competencies in this way may contribute to safer and more autonomous care within the facility.

Thirdly, clinical evaluation by a GP prior to transfer should be encouraged wherever feasible, as this is associated with a lower likelihood of avoidable admissions [20, 29, 41]. Early assessment may aid in distinguishing between cases that require ED admission and those that could be managed within the NH.

Fourthly, advance care planning should be systematically promoted and documented to ensure that medical decisions are aligned with NHRs' individual preferences and care goals. The proactive establishment of advance care plannings has the potential to prevent transfers that do not reflect NHRs’ wishes, particularly in the context of multimorbidity or end-of-life care [9, 29].

Moreover, the establishment of geriatric outreach services or in-home consultations may provide valuable support in managing clinically complex, yet non-urgent, situations that might otherwise prompt an ED referral [29, 41]. Dai et al. reported a 36.1% reduction in hospital admissions after implementing an acute geriatric outreach service delivering hospital-level interventions [49].

Finally, the development of direct admission pathways to geriatric or others wards, bypassing the ED, could reduce the risks associated with ED stays while ensuring that necessary hospital care is provided efficiently and appropriately [52, 53].

Nevertheless, it is essential to recognise that NHs are, first and foremost, places of residence rather than medical institutions. Any proposed expansion of healthcare services within these settings must be carefully balanced against the need to preserve NHRs' autonomy, comfort, and quality of life [16]. Interventions should therefore be evaluated not only in terms of clinical efficacy but also with regard to their resource implications and acceptability to both staff and NHRs [10].

## Conclusion

This study has quantified and provided valuable insights into the issue of avoidable ED admissions in Belgium. It also presents a specific tool for identifying and quantifying retrospectively avoidable admissions. The lack of significant differences in resident characteristics between avoidable and non-avoidable admissions suggests that system-level factors may play a more decisive role. In this perspective, concrete avenues for improvement are proposed, aiming to better target interventions.These initiatives must be carefully designed to enhance care quality without compromising NHR safety.

## Supplementary Information

Below is the link to the electronic supplementary material.

Supplementary file1 (DOCX 48 KB)

Supplementary file2 (DOCX 74 KB)

## Data Availability

Due to the institutional ethics approval granted, the dataset generated and analysed during this study is not publicly available. However, further information can be obtained from the corresponding author upon reasonable request. The research protocol is also available upon request from the corresponding author.
