# Correction: Avoidable emergency department admissions among nursing home residents – insights from a retrospective study

**DOI:** 10.1007/s41999-025-01346-1

**Published:** 2025-12-04

**Authors:** Julie Merche, Henri Thonon, François-Xavier Sibille, Julie Gabriel, Elise Simonin, Didier Schoevaerdts, Thérèse Van Durme, Marie de Saint-Hubert

**Affiliations:** 1 Department of Geriatric Medicine, Centre Hospitalier Universitaire UCL Namur, Namur, Belgium; 2 UCLouvain, Institute of Health and Society, Brussels, Belgium; 3 Emergency Department, Centre Hospitalier Universitaire UCL Namur, Namur, Belgium; 4 UCLouvain, Institute of Experimental and Clinical Research, Brussels, Belgium; 5 Department of Geriatric Medicine, Centre Hospitalier Universitaire Vaudois, Lausanne, Switzerland; 6 UCLouvain, Faculty of Public Health, Brussels, Belgium

**Correction: European Geriatric Medicine** 10.1007/s41999-025-01264-2

In the originally published version of this article, the median values reported in the Results section were incorrectly displayed as reference numbers due to a formatting error during typesetting. The correct presentation of the medians is now provided in the updated version of the article. This error does not affect the results or conclusions of the study.

The Original Article has been corrected.

