# Supplementary material for: Avoidable emergency department admissions among nursing home residents – insights from a retrospective study
Source: Eur Geriatr Med. 2025 Jul 3;17(1):347–61. doi: 10.1007/s41999-025-01264-2 (PMC12946282; doi:10.1007/s41999-025-01264-2)
Supplement: Supplementary file 1 — Supplementary file1 (DOCX 48 KB) [file 41999_2025_1264_MOESM1_ESM.docx]

# **Additional file 1: criteria used to define avoidability**

| Inclusion and exclusion criteria | Rationale |
| --- | --- |
| Hospitalization required following ED assessment | The avoidability of a visit appears strongly linked to the absence of hospitalization; for instance, Morphet et al. reported that one-third of NHRs who returned home after their visit could have avoided the emergency transfer [1], while Codde et al. estimated this proportion at 69% [2].  Burke explored emergency visits that did not lead to hospitalization, describing them as potentially avoidable, although acknowledging that not all such visits can be prevented [3].  Similarly, examiners often deemed transfers inappropriate when the NHR ’s primary complaint did not necessarily justify hospitalization [4].  Moreover, avoidable cases frequently correspond to emergency visits without subsequent admission [5]. |
| Presenting complaint: acute condition justifying presentation to the emergency department  (e.g., diagnosed fracture, suspected stroke, active bleeding, chest pain, etc.) | These presenting complaints justify seeking emergency care. Regardless of the final diagnosis, if the initial complaint is categorized as urgent during triage, the admission is not considered avoidable. |
| Severe clinical parameters | Low-acuity visits are associated with lower-severity triage scores [1, 6-9]. Admissions resulting in discharge to home are associated with normal clinical parameters [3]. We used the National Early Warning Score with the cut off of 2 points for each parameter [10]. |
| Diagnostic work-up performed:  CT scan, ultrasound, or X-ray outside regular hours; specialist consultation required | Visits that did not result in hospitalization often involved no diagnostic testing [3], and utilized fewer resources [9]. These visits frequently consisted of evaluations with basic laboratory tests or radiographic imaging conducted during regular working hours [1, 11]. |
| Low-acuity discharge diagnoses (non-severe outcomes) | Minor falls are frequently cited as a common cause of potentially avoidable admissions [12, 13]. Patients admitted for injuries are more likely to be discharged home than to require hospitalization [3]. Avoidable admissions among NHRs primarily involve minor complaints such as urinary tract infections, incontinence, skin lacerations, sprains, and pain [1, 2, 5].  Low-acuity visits are mostly related to fall-related injuries but also involve the adjustment or management of implanted devices and pain [7, 8, 14]. Suturing can be performed by GP in primary care settings [15]. |

NHR: nursing home resident. ED: emergency department. GP: general practitioner. CT: Computed tomography.

## **References**

1. Morphet J, Innes K, Griffiths DL, Crawford K, Williams A: Resident transfers from aged care facilities to emergency departments: can they be avoided? Emerg Med Australas 2015, 27(5):412-418. <https://doi.org/10.1111/1742-6723.12433>

2. Codde J, Frankel J, Arendts G, Babich P: Quantification of the proportion of transfers from residential aged care facilities to the emergency department that could be avoided through improved primary care services. Australas J Ageing 2010, 29(4):167-171. <https://doi.org/10.1111/j.1741-6612.2010.00496.x>

3. Burke RE, Rooks SP, Levy C, Schwartz R, Ginde AA: Identifying Potentially Preventable Emergency Department Visits by Nursing Home Residents in the United States. J Am Med Dir Assoc 2015, 16(5):395-399. <https://doi.org/10.1016/j.jamda.2015.01.076>

4. Saliba D, Kington R, Buchanan J, Bell R, Wang M, Lee M *et al*: Appropriateness of the decision to transfer nursing facility residents to the hospital. J Am Geriatr Soc 2000, 48(2):154-163. <https://doi.org/10.1111/j.1532-5415.2000.tb03906.x>

5. Popejoy LL, Vogelsmeier AA, Alexander GL, Galambos CM, Crecelius CA, Ge B *et al*: Analyzing Hospital Transfers Using INTERACT Acute Care Transfer Tools: Lessons from MOQI. J Am Geriatr Soc 2019, 67(9):1953-1959. <https://doi.org/10.1111/jgs.15996>

6. Briggs R, Coughlan T, Collins R, O'Neill D, Kennelly SP: Nursing home residents attending the emergency department: clinical characteristics and outcomes. QJM 2013, 106(9):803-808. <https://doi.org/10.1093/qjmed/hct136>

7. Cetin-Sahin D, Karanofsky M, Cummings GG, Vedel I, Wilchesky M: Measuring Potentially Avoidable Acute Care Transfers From Long-Term Care Homes in Quebec: a Cross Sectional Study. Can Geriatr J 2023, 26(3):339-349. <https://doi.org/10.5770/cgj.26.620>

8. Gruneir A, Bell CM, Bronskill SE, Schull M, Anderson GM, Rochon PA: Frequency and pattern of emergency department visits by long-term care residents--a population-based study. J Am Geriatr Soc 2010, 58(3):510-517. <https://doi.org/10.1111/j.1532-5415.2010.02736.x>

9. Zúñiga F, Gaertner K, Weber-Schuh SK, Löw B, Simon M, Müller M: Inappropriate and potentially avoidable emergency department visits of Swiss nursing home residents and their resource use: a retrospective chart-review. BMC Geriatr 2022, 22(1):659. <https://doi.org/10.1186/s12877-022-03308-9>

10. Guan G, Lee CMY, Begg S, Crombie A, Mnatzaganian G: The use of early warning system scores in prehospital and emergency department settings to predict clinical deterioration: A systematic review and meta-analysis. PLoS One 2022, 17(3):e0265559. <https://doi.org/10.1371/journal.pone.0265559>

11. Dwyer R, Gabbe B, Stoelwinder JU, Lowthian J: A systematic review of outcomes following emergency transfer to hospital for residents of aged care facilities. Age Ageing 2014, 43(6):759-766. <https://doi.org/10.1093/ageing/afu117>

12. Lamb G, Tappen R, Diaz S, Herndon L, Ouslander JG: Avoidability of hospital transfers of nursing home residents: perspectives of frontline staff. J Am Geriatr Soc 2011, 59(9):1665-1672. <https://doi.org/10.1111/j.1532-5415.2011.03556.x>

13. Unroe KT, Caterino JM, Stump TE, Tu W, Carnahan JL, Vest JR *et al*: Long-Stay Nursing Facility Resident Transfers: Who Gets Admitted to the Hospital? J Am Geriatr Soc 2020, 68(9):2082-2089. <https://doi.org/10.1111/jgs.16633>

14. Aryal K, Mowbray FI, Strum RP, Dash D, Tanuseputro P, Heckman G *et al*: Examining the "Potentially Preventable Emergency Department Transfer" Indicator Among Nursing Home Residents. J Am Med Dir Assoc 2023, 24(1):100-104.e102. <https://doi.org/10.1016/j.jamda.2022.10.006>

15. Heinold S, Fassmer AM, Schmiemann G, Hoffmann F: Characteristics of outpatient emergency department visits of nursing home residents: an analysis of discharge letters. Aging Clin Exp Res 2021, 33(12):3343-3351. <https://doi.org/10.1007/s40520-021-01863-6>
