# Supplementary material for: Avoidable emergency department admissions among nursing home residents – insights from a retrospective study
Source: Eur Geriatr Med. 2025 Jul 3;17(1):347–61. doi: 10.1007/s41999-025-01264-2 (PMC12946282; doi:10.1007/s41999-025-01264-2)
Supplement: Supplementary file 2 — Supplementary file2 (DOCX 74 KB) [file 41999_2025_1264_MOESM2_ESM.docx]

# **Additional file 2: Distribution of the number of emergency department admissions per nursing home resident over the study year**

**
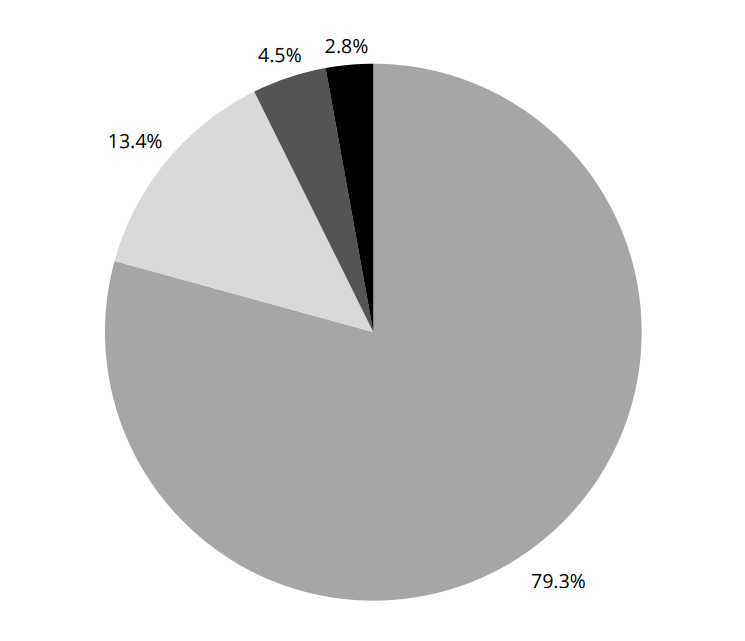
**


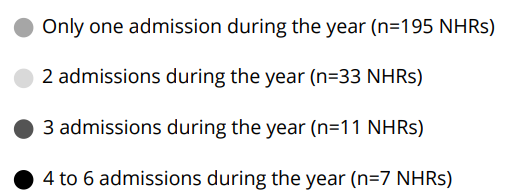


NHRs : nursing home residents
